# Hypoxia promotes the metastasis of pancreatic cancer through regulating NOX4/KDM5A-mediated histone methylation modification changes in a HIF1A-independent manner

**DOI:** 10.1186/s13148-021-01016-6

**Published:** 2021-01-26

**Authors:** Hongzhen Li, Chunyan Peng, Chenhui Zhu, Shuang Nie, Xuetian Qian, Zhao Shi, Mengyue Shi, Yan Liang, Xiwei Ding, Shu Zhang, Bin Zhang, Xihan Li, Guifang Xu, Ying Lv, Lei Wang, Helmut Friess, Bo Kong, Xiaoping Zou, Shanshan Shen

**Affiliations:** 1 Department of Gastroenterology, Nanjing Drum Tower Hospital, The Affiliated Hospital of Nanjing University Medical School, No. 321 Zhongshan Road, Nanjing, 210008 Jiangsu China; 2 Department of Gastroenterology, Nanjing Drum Tower Hospital, Clinical College of Nanjing Medical University, Nanjing, Jiangsu China; 3 Department of Pathology, East Region Military Command General Hospital, Nanjing, Jiangsu China; 4 Department of Surgery, Klinikum Rechts Der Isar, School of Medicine, Technical University of Munich (TUM), Munich, Germany

**Keywords:** Hypoxia, Histone methylation modification, Epithelial-to-mesenchymal transition, NOX4, SNAIL1

## Abstract

**Background:**

Hypoxia is a characteristic of the tumor microenvironments within pancreatic cancer (PC), which has been linked to its malignancy. Recently, hypoxia has been reported to regulate the activity of important carcinogenic pathways by changing the status of histone modification. NOX4, a member of NADPH oxidase (NOX), has been found to be activated by hypoxia and promote cancer progression in several cancers. But whether it is involved in the epigenetic changes of tumor cells induced by hypoxia is still unclear, and its biological roles in PC also need to be explored.

**Methods:**

A hypoxic-related gene signature and its associated pathways in PC were identified by analyzing the pancreatic cancer gene expression data from GEO and TCGA database. Candidate downstream gene (NOX4), responding to hypoxia, was validated by RT-PCR and western blot. Then, we evaluated the relationship between NOX4 expression and clinicopathologic parameters in 56 PC patients from our center. In vitro and in vivo assays were preformed to explore the phenotype of NOX4 in PC. Immunofluorescence, western blot and chromatin immunoprecipitation assays were further applied to search for a detailed mechanism.

**Results:**

We quantified hypoxia and developed a hypoxia signature, which was associated with worse prognosis and elevated malignant potential in PC. Furthermore, we found that NADPH oxidase 4 (NOX4), which was induced by hypoxia and upregulated in PC in a HIF1A-independent manner, caused inactivation of lysine demethylase 5A (KDM5A), increased the methylation modification of histone H3 and regulated the transcription of EMT-associated gene_ snail family transcriptional repressor 1 (SNAIL1). This served to promote the invasion and metastasis of PC. NOX4 deficiency repressed hypoxia-induced EMT, reduced expression of H3K4ME3 and impaired the invasion and metastasis of PC cells; however, knockdown of KDM5A reversed the poor expression of H3KEME3 induced by NOX4 deficiency, thereby promoting EMT.

**Conclusions:**

This study highlights the prognostic role of hypoxia-related genes in PC and strong correlation with EMT pathway. Our results also creatively discovered that NOX4 was an essential mediator for hypoxia-induced histone methylation modification and EMT in PC cells.

## Introduction

Hypoxia is a vital feature of the tumor microenvironment, including pancreatic cancer (PC) [1, 2]. Increased desmoplasia and resulting insufficient perfusion are important causes of hypoxia in PC [3, 4]. Triggered by hypoxia, tumor cells activate a variety of molecular pathways in order to adapt to environmental change [5]. Epithelial-to-mesenchymal transition (EMT) is a process of transforming tumor cells from epithelial to mesenchymal cell types, which could be induced by hypoxia, contributing to tumor invasion and metastasis [6, 7]. In this regard, most studies proposed that hypoxia-inducible factor 1 subunit alpha (HIF1A) is held accountable for the induction of EMT [8, 9]. However, this view has been updated by the latest studies showing that the change of histone modifications under hypoxia occurred more rapidly than HIF1A induction. This rapid response in histone modification is able to induce EMT process in Hela cells [10, 11]. However, the mechanism of hypoxia-induced histone methylation and its association with EMT in PC is not yet clear.

It is generally believed that our cells were capable of sensing the ambient oxygen concentration and responding to hypoxia. Hypoxia-induced ROS production has been reported to link the stabilization of HIF1A and activation of redox-sensitive K + channels, which was thought to be an important way of oxygen sensing [12]. And NADPH oxidase (NOX), a main reactive oxygen species (ROS) producer, can be activated in response to hypoxia [13, 14]. Different from the inevitable physiological “leakage” of the mitochondrial respiratory chain during normal function, the enzymes’ NADPH oxidase is the primary source of non-mitochondrial ROS production, and more importantly, they generate ROS in a regulated manner [15]. Hence, the NADPH oxidase has been suggested as a possible cellular oxygen sensor [16]. And also, NADPH oxidase has been reported to induce EMT in breast cancer cells and hepatocellular carcinoma [17, 18]. Therefore, NADPH oxidase may be involved in hypoxia-induced malignant biological behavior of PC.

Histone H3 lysine 4 trimethylation (H3K4me3) at the promoter region of genes fulfils a key role in the regulation of transcriptional activation. Lysine demethylase 5A (KDM5A), a member of histone demethylase and the most abundant KDM5 family member, is capable of removing tri- and dimethyl marks of Histone H3 lysine 4, which has been reported to inhibit the migration and invasion of glioma cells [19]. More importantly, KDM5A is believed to be sensitive to hypoxia at the cellular level and controls a variety of cellular responses [10]. Since the change of oxidative stress state is an important process of hypoxia, we suspect that oxidative stress may be related to the regulation of histone modification, especially the regulation of histone demethylase.

In this study, we took advantage of GEO and TCGA databases to establish a list of hypoxia-associated genes. Further analysis uncovered a prognostic value of hypoxia-related gene sets, which is also associated with EMT-related pathways. Then, we identified NOX4, the enzyme activated by hypoxia in PC, activated EMT pathway by regulating inactivation of KDM5A and by modifying chromatin methylation modification.

## Materials and methods

### Analysis of pancreatic cancer gene expression data from GEO and TCGA database

The hypoxia-related gene expression signature consisted of 200 genes and EMT-related gene expression signature consisted of 200 genes were obtained from the gene set of HALLMARK_HYPOXIA and HALLMARK_EPITHELIAL_MESENCHYMAL_TRANSITION in The Molecular Signatures Database (MSigDB, https://www.gsea-msigdb.org/). Microarray gene expression data from GSE15471 and GSE16515 were used to screen for genes that were highly expressed in PC tissues (fold-change > 2 and adjusted p value < 0.05) and then merged with 200 hypoxia-related genes. We got 30 highly expressed hypoxia-related genes in pancreatic cancer tissues. Level3 TCGA RSEM Gene expression data and clinical information for pancreatic cancer were downloaded from UCSC (https://xena.ucsc.edu/public). Then, we calculated the hypoxia score of TCGA samples using the 30 genes as previously described [20]. Briefly, for each gene, samples with the top 50% of expression value were given a score of + 1, and samples with the bottom 50% of expression value were given a score of – 1. The hypoxia score in each sample was the sum of the scores of 30 genes (Additional file 4: Table S1). Similarly, EMT score of TCGA samples was calculated using 200 EMT-related genes using the same method (Additional file 5: Table S2). TCGA subtype defined by Bailey et al. [21], Collisson et al. [22], and Moffitt et al. [23] was derived from the most recent TCGA pancreatic adenocarcinoma subclassification [24].

### Functional analysis and GSVA

Pearson correlation analysis was used to find genes related to hypoxia scores (Additional file 6: Table S3). GSVA was applied to calculate the signature enrichment scores of individual samples from TCGA as previously described. The R package “clusterProfiler” [25] was applied for the Gene Ontology (GO) analysis of genes positively related to hypoxia score (p value < 0.05). Hallmark gene sets were enriched using Metascape (p value < 0.05) [26].

### Tumor samples

Fifty-six PC formalin-fixed paraffin-embedded (FFPE) samples were obtained from the Affiliated Drum Tower Hospital of Nanjing University Medical School (Nanjing, China) between January 2007 and August 2013. Frozen specimens of 6 PC patients and 6 benign pancreatic lesions were collected from the same hospital between 2018 and 2019. The experimental study was approved by the Affiliated Drum Tower Hospital of Nanjing University Medical School, and the informed consent forms were obtained from patients enrolled in this study.

### Cell culture and treatment

Human pancreatic cancer cell lines HPAC and Panc1 were a gift from the Technical University of Munich, Germany. All cell lines used in this study are considered to be identical to the reference cell line in the Cell Bank STR database, as the STR profile yields a 100% match. All cell lines were cultured in DMEM medium (Wisent Inc, Montreal, Canada) with 10% FBS (Biological Industries, Beit HaEmek, Israel) under 5% CO_2_ at 37 °C. For hypoxic experiments, cells were incubated in an incubator with a humidified atmosphere of 1% O2, 5% CO2 and 94% N2. Other interventions for cells are shown in Additional file 1: supplementary materials.

### NOX4 knockdown and overexpress

NOX4 short-hairpin RNAs (shRNAs) or scrambled control shRNA was designed, synthesized and packaged into lentivirus particles (Corues Biotechnology, Nanjing, China). Cells were plated into six-well plates (3 × 10^5^ cells per well). Before transfection, the culture medium was replaced with DMEM with 10%FBS and 1 µg/mL Polybrene (GeneChem, Shanghai, China). Infectious lentivirus particles were harvested for 72 h after transfection.

To establish NOX4-overexpressed HPAC cells, HPAC cells were transfected with NOX4 Human Tagged ORF Clone (RC208007) and the pcmv6 empty vector (OriGene, MD, USA) using lipo3000 (Thermo Fisher Scientific, MA, USA). After 72 h, cells were treated with G418 (Sigma-Aldrich Corp., MO, USA) to select stably transfected clones.

### RNA extraction and RT-PCR

Total RNA was isolated from cells using RNAiso Plus Reagent (Takara, Kusatsu, Japan). RT reactions were performed using the PrimeScript™ RT Master Mix (Takara). Then, the quantification of mRNA expression was performed using SYBR^®^ Advantage^®^ qPCR Premix (Takara) in a total reaction volume of 20 µl according to the manufacturer's instructions. The reaction was performed using the LightCycler^®^ 96 system (Roche Diagnostics, Basel, Switzerland). ACTB was used as an internal control. The primer sequences used are shown in Additional file 1: supplementary material.

### Immunoblot analysis

Cell or tissue homogenate was used for immunoblot analysis. Specific information is described in Additional file 1: supplementary materials.

### Immunohistochemistry

Paraffin sections of tissues from 56 PC patients were used for immunohistochemical detection of NOX4 expression. The detailed protocol is presented in Additional file 1: supplementary material.

### Immunofluorescence

Cells or frozen tissue sections  (15um thick) were fixed with paraformaldehyde, permeabilized with 0.2% Triton X-100, blocked with 2% BSA in PBS and incubated with NOX4, CDH2, VIM, H3K4ME3 antibodies overnight at 4 °C. Then, the sections and cells were incubated with secondary antibody (1:500, Additional file 1: supplementary material) for 2 h, counterstained with DAPI (Beyotime) and visualized by a fluorescence microscope (Olympus, Tokyo, Japan).

### Migration and invasion assays

Migration and invasion assays were proceeded in a transwell chamber (Corning, NY, USA) with (invasion) or without (migration) Matrigel matrix (Corning) in a 24-well plate. 5 × 10^6^ cells resuspended in serum-free DMEM were added in the up chamber, and DMEM medium with 20% FBS was added in the bottom chamber. Then, cells were fixed with 4% paraformaldehyde and stained with 1% crystal violet. Cells were imaged and counted using a 20 × microscope.

### Experimental mice

Four-week-old male BALB/c nude mice (weighing 16–18 g) were purchased from Changzhou Cavens Experimental Animal Co. Ltd (Changzhou, China). For lung-metastasis xenografts, the mice were randomly divided into three groups (control, shNOX4#1, SHNOX4#2). 1 × 10^6^ HPAC cells with or without NOX4 knockdown suspended in 100 µL cold PBS were injected into the lateral tail vein (4foreachgroup). After one month, all mice were killed, and lung tissues were fixed with 4% paraformaldehyde. H&E staining was used to evaluate the proportion of metastatic lesions. All animals were approved by the Ethics Committee of Nanjing Drum Tower Hospital. All animals used in this study were treated humanely and followed guidelines set by the Animal Care Committee. The study was approved by the Ethics Review Committee for Animal Experimentation at Nanjing Drum Tower Hospital (Nanjing, China).

### CHIP-PCR

Chromatin immunoprecipitation (CHIP)-PCR was performed to analyze the effect of NOX4 on the binding of H3K4ME3 to the SNAIL1 promoter sequence. A detailed information is presented in Additional file 1: supplementary material.

### Statistical analysis

All bioinformatics analyses were performed using R (https://www.r-project.org/) and RStudio software (B Corps™, DE, USA). All statistical analysis was performed using SPSS version 24.0 for Windows (SPSS Inc., an IBM company, Chicago, IL, USA) and GraphPad Prism v6.0 (GraphPad Inc., La Jolla, CA, USA) software. All data were reported as the mean ± SD. The differences between two groups were analyzed using T-test, and the differences among multiple groups were analyzed using one-way ANOVA or two-way ANOVA followed by Tukey’s test. Correlations between two groups were analyzed by the Pearson's rank-order method. Kaplan–Meier curve (log-rank tests) was used to determine any significant associations of patient outcome and hypoxia score or NOX4. Cox proportional hazard models and partial correlation analysis were used to evaluate the factors associated with survival and EMT score using forward: LR strategy. Two-sided P value of < 0.05 was considered statistically significant.

## Result

### Evaluation of hypoxia-related gene expression in PC

**Fig. 1:**
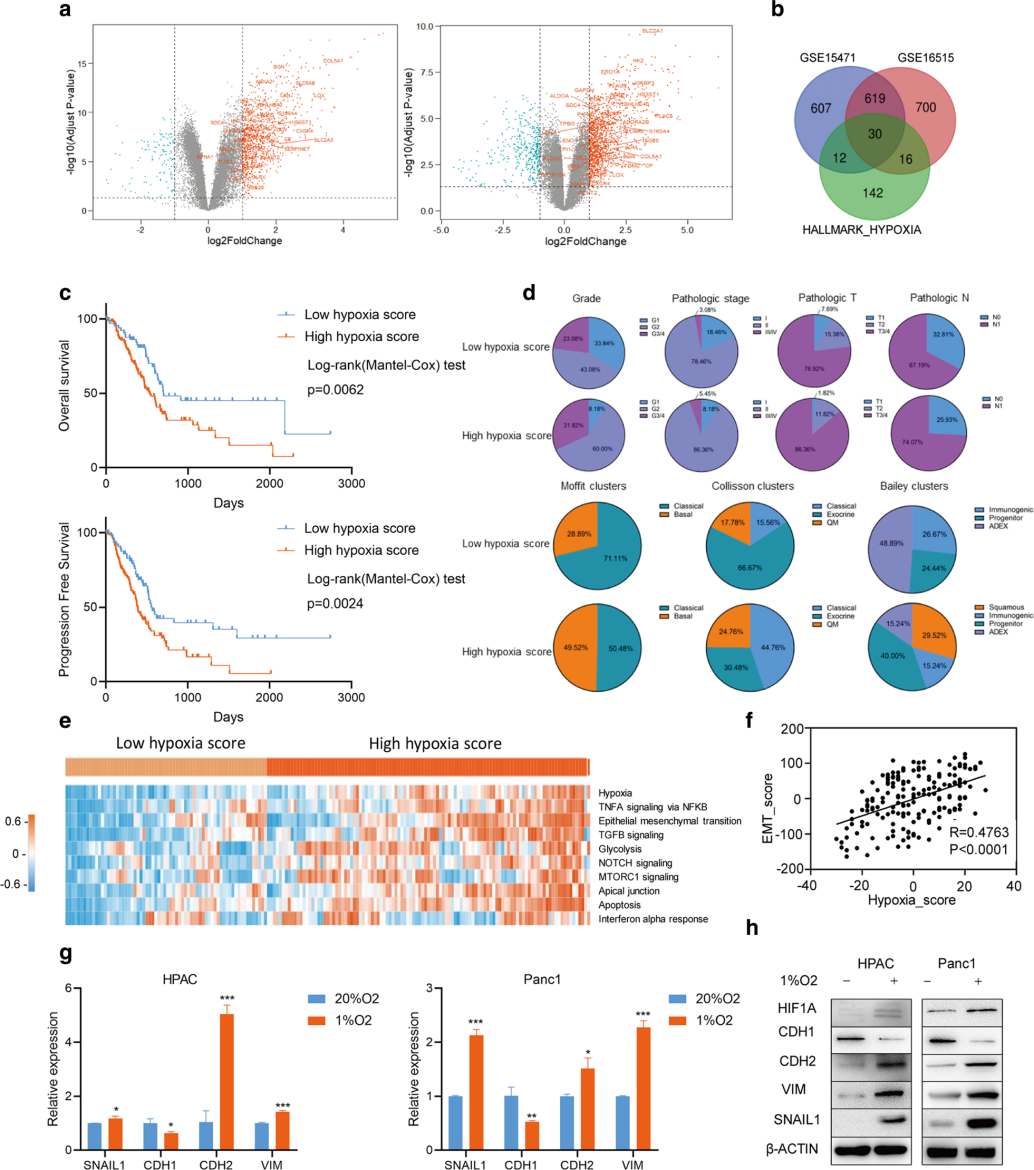
Hypoxia-related gene expression was enriched in a subset of pancreatic cancer samples and correlated with EMT pathway. **a** The volcano diagrams showed differentially expressed mRNAs between pancreatic cancer tissue and normal tissue samples from GSE15471 (left panel) and GSE16515 (right panel). Hypoxia-related genes were highlighted using gene name. **b** Venn diagram showed that 30 hypoxia-related genes were both upregulated in tumor tissues in 2 databases. **c** Overall survival and progression-free survival between the high hypoxia score group and low hypoxia score group. **d** Pie charts showing proportions of the clinical parameters and molecular subtypes between high hypoxia score group and low hypoxia score group in samples of pancreatic cancer from TCGA. **e** The heat map showed the top10 gene sets after GSVA analysis. **f** Pearson’s correlation analyses between hypoxia score and EMT score. **g** The mRNA and **h** protein expression of EMT markers after 1%O_2_ treatment for 24 h were assessed by qRT-PCR. *p < 0.05, **p < 0.01, ***p < 0.001

First, we quantified hypoxia-related gene expression in GSE15471 and GSE16515 using 200 genes from hallmark HYPOXIA gene set (Molecular Signatures Database [MsigDB]). This analysis identified 30 shared hypoxia-related genes that were overexpressed in PCs, compared to normal controls in both databases (Fig. 1a, b). Additional file 2: Figure S1A shows the expression of these 30 genes across PAAD tumor samples in TCGA dataset. Then, we used these 30 genes as hypoxia-related gene signature, and a hypoxia score was calculated for each individual sample according to the expression levels of these genes (described in the method part, Additional file 4: Table S1). Next, we explored the prognostic significance of hypoxia-related gene expression in TCGA PAAD dataset. Tumors with high hypoxia score values had a worse overall survival (OS) and progression-free survival (PFS) than those with low hypoxia score values (Fig. 1c). And also, hypoxic tumors were more likely to have a higher grade and pathologic stage (Fig. 1d). In a multivariate Cox regression model including stage, grade, residual tumor and hypoxia score, which were significantly related to the prognosis in the univariate analysis, hypoxia score was an independent predictor of OS (p = 0.038, Table 1) and PFS (p = 0.032, Table 2). To verify whether hypoxia scores are related to specific molecular subtypes, we grouped TCGA PAAD samples by the four-group classification of Bailey et al. [21], the three-group classification of Collisson et al. [22], and the two-group classification of Moffitt et al. [23]. This analysis revealed that the subtypes with unfavorable prognosis (squamous in Bailey clusters, quasimesenchymal (QM) in Collisson clusters and basal-like in Moffitt clusters) were more likely to have a higher hypoxia score (Fig. 1d).

**Table 1:** Univariate and multivariate analysis for overall survival (OS)

	Univariate			Multivariate			
	Hazard ratio	95%CI	p value	Hazard ratio	95%CI	p value	
Gender	0.8049	0.5326–1.216	0.2889				
Hypoxia score	1.834	1.216–2.767	0.0062*	1.654	1.028–2.662		0.038*
Grade				–	–		0.149
G1			Reference				
G2	2.004	1.160–3.463	0.0283*				
G3/G4	2.401	1.309–4.405	0.0089*				
Stage				–	–		0.142
I			Reference				
II	2.271	1.293–3.989	0.0292*				
III/IV	1.029	0.2640–4.010	0.9665				
Location (head vs body and tail)	0.5812	0.3501–0.9649	0.0732				
Residual tumor	1.632	1.020–2.609	0.0247*	1.568	1.005–2.447		0.048*

*Statistically significant in the univariate and multivariate analysis

**Table 2:** Univariate and multivariate analysis for progression-free survival (PFS)

	Univariate			Multivariate			
	Hazard ratio	95%CI	p value	Hazard ratio	95%CI	p value	
Gender	0.9699	0.6583–1.429	0.8769				
Hypoxia score	1.861	1.264–2.741	0.0024*	1.646	1.043–2.597		0.032*
Grade				–	–		0.139
G1			Reference				
G2	1.623	1.010–2.774	0.0283*				
G3/G4	2.124	1.199–3.762	0.0089*				
Stage				–	–		0.189
I			Reference				
II	2.745	1.628–4.630	0.0292*				
III/IV	1.911	0.5360–6.816	0.2483				
Location (head vs body and tail)	0.5963	0.3738–0.9511	0.057				
Residual tumor	2.289	1.444–3.628	0.0001*	1.988	1.303–3.035		0.001*

*Statistically significant in the univariate and multivariate analysis

### Epithelial–mesenchymal transition was induced in hypoxia samples

To determine whether specific cancer-promoting pathways were shared across the hypoxia PC samples, we performed gene set variation analysis (GSVA) [27], which showed that the following GSVA signatures were upregulated in the hypoxia tumor samples: TNFA signaling via NFKB, epithelial–mesenchymal transition, TGFB signaling, glycolysis, NOTCH signaling, etc. (Fig. 1e). We also analyzed the biological processes and pathways that were positively correlated with hypoxia score through calculating the Pearson correlation coefficient between each hypoxia score and all the other genes in TCGA PAAD samples (Additional file 6: Table S3, R ≥ 0.05, p < 0.05). The GO analysis showed that the biological processes such as extracellular structure organization and extracellular matrix organization were significantly enriched (p < 0.05, Additional file 2: Figure S1B), and the Hallmark gene sets analysis identified EMT pathway is the most enriched one (p < 0.05, Additional file 2: Figure S1C). Additional file 2: Figure S1D shows that the expression of EMT markers consisting of 200 genes increases along with hypoxia score. As above mentioned, we then calculated an EMT score for each individual sample and tested whether it was correlated with the hypoxia score. This analysis revealed that EMT and hypoxia score were strictly correlated with each other (Fig. 1f). Then, we used stage, grade and residual tumor as control variables for partial correlation analysis, and the results showed that the hypoxia score was positively correlated with the EMT score (Table 3). To experimentally validate this, we tested the expression of EMT-related genes in two PC cell lines treated with/without hypoxia (1% O_2_). This analysis revealed that the mRNA and protein expression of vimentin (VIM), cadherin 2 (CDH2) and SNAIL1 increased in HPAC and Panc1 cell lines upon hypoxia exposure for 24 h. Accordingly, the mRNA and protein expression of cadherin 1 (CDH1) decreased in both cell lines (Fig. 1g, h).

**Table 3:** Pearson correlation and partial correlation between clinical factors and EMT score

	Pearson correlation		Partial correlation	
	Coefficient	p value	Coefficient	p value
Hypoxia score	0.476	< 0.0001	0.452	< 0.0001
Grade	0.141	0.062	Control	
Stage	0.174	0.021	Control	
Residual tumor	0.004	0.958	Control	

### The expression of NOX4 induced by hypoxia contributed to the EMT process in PC cells

**Fig. 2:**
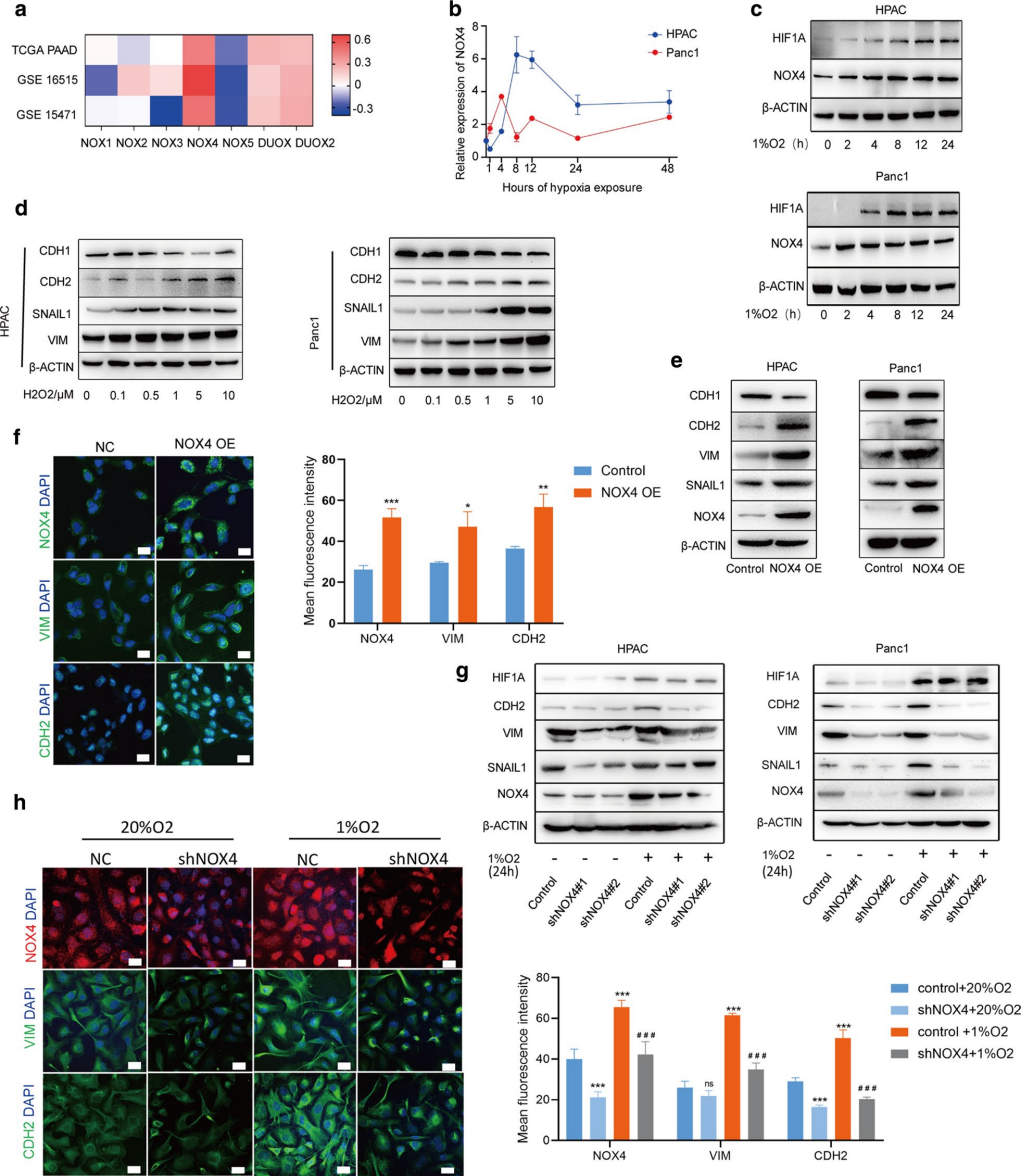
EMT induced by hypoxia in pancreatic cancer cells was dependent on NOX4 expression. **a** The heat map showed Pearson’s correlation analyses between hypoxia score and NADPH oxidase. **b** The mRNA and **c** protein expression of NOX4 after time gradient of hypoxia treatment in HPAC and Panc1 cells. **d** The protein expression of CDH1, CDH2, VIM and SNAIL1 after the concentration gradient of H_2_ O_2_ treatment. **e** The protein expression of CDH1, CDH2, VIM and SNAIL1 after NOX4 overexpression. **f** The expression and quantification of signal intensities of NOX4, VIM, CDH2 in HPAC cells after NOX4 overexpression were determined by immunofluorescence. Bar = 20 μm. **g** The protein expression of CDH1, CDH2, VIM and SNAIL1 after NOX4 knockdown and hypoxia treatment. **h** The expression and quantification of signal intensities of NOX4, VIM, CDH2 in HPAC cells after NOX4 knockdown and hypoxia treatment were determined by immunofluorescence. Bar = 20 μm. ns, no significance, *p < 0.05, **p < 0.01, ***p < 0.001

Since hypoxia was described to aggravating the oxidative stress process, which was implicated with EMT process, we first evaluated the correlation between hypoxia-related expression and NOX family members, which are responsible for generating non-mitochondrial ROS [28]. This analysis revealed a significant correlation between NOX4 expression and hypoxia score (Fig. 2a). We used the median value to distinguish groups with high and low expression of NOX4 in TCGA samples and carried out a GSEA analysis. Importantly, this analysis identified EMT and hypoxia pathways were significantly enriched in samples with high expression of NOX4 (with a cutoff for FDR 5% and p value < 0.01) (Additional file 3: Figure S2A).

Further, we detected the location of NOX4 in the bulk tissues of PC patients. NOX4 was mainly expressed in epithelial cells (Additional file 3: Figure S2B). To further verify the expression of NOX4 after hypoxia, we examined the mRNA and protein expression of NOX4 after hypoxia exposure for 0–24 h. We found that NOX4 expression increases at a very early stage after hypoxia exposure (Fig. 2b, c). We also found that the level of ROS increased steadily after hypoxia exposure in HPAC cells (Additional file 3: Figure S2C). Since superoxide is generated from NOX4 and quickly converts to H2O2 through dismutation [29], we hypothesized that H2O2 was partially responsible for NOX4 and hypoxia-induced EMT phenotype. Indeed, H2O2 activated an EMT program in HAPC and Panc1 cells in a dose-dependent manner (Fig. 2d). In line, the level of ROS increased significantly when NOX4 was overexpressed in HAPC and Panc1 cells (Additional file 3: Figure S2D). Correspondingly, EMT process is also activated in these NOX4-overexpressing cells (Fig. 2e, f). When NOX4 was knocked down using two short-hairpin RNAs (shNOX4#1 and shNOX4#2) in HPAC and Panc1 cells, the hypoxia-induced EMT process was partially compromised (Fig. 2g, h). Thus, NOX4 plays an important role in the process of EMT induced by hypoxia in PC cells.

### NOX4 was highly expressed by PDAC and promoted the invasion and metastasis of PC cells

**Fig. 3:**
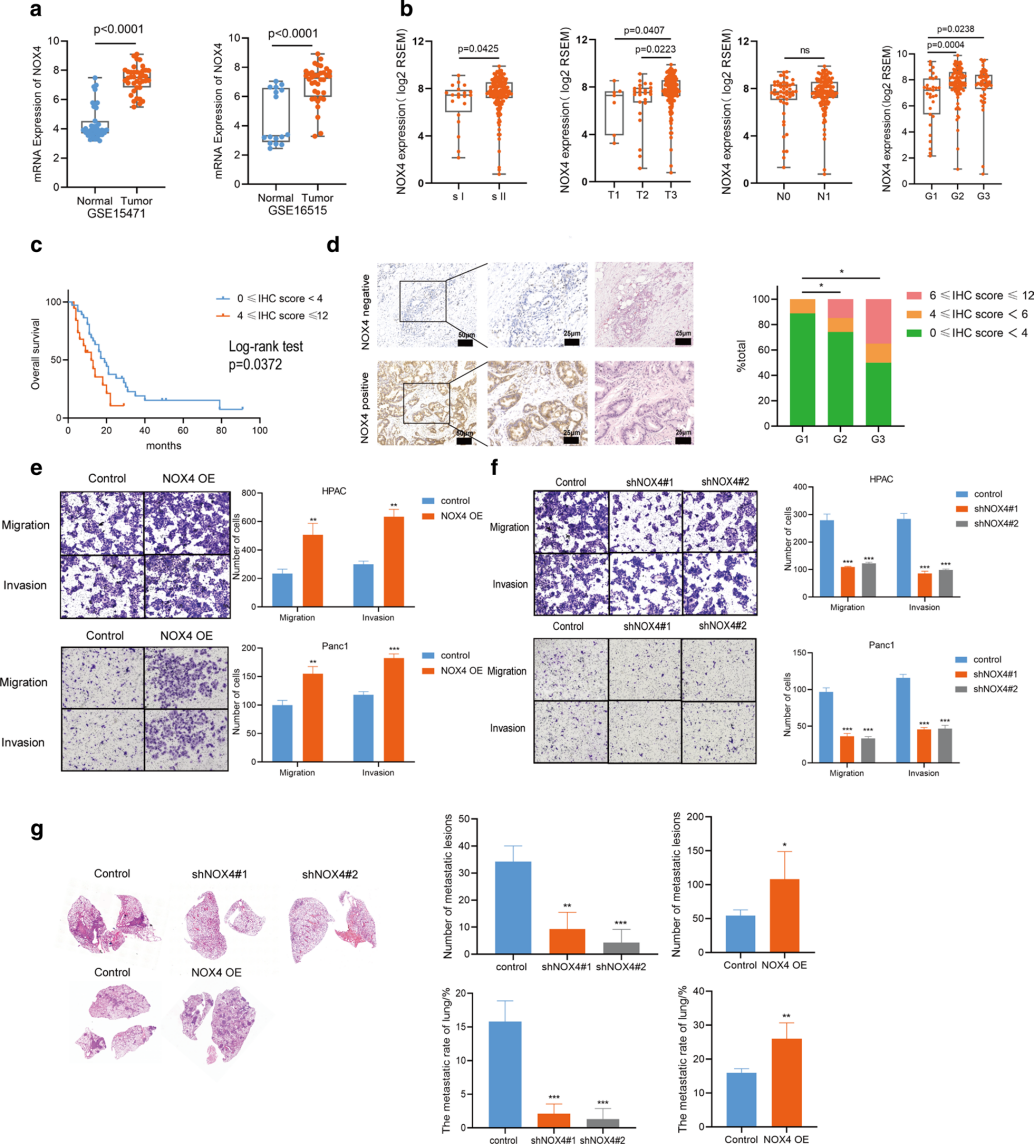
NOX4 was upregulated in pancreatic cancer and promoted the metastasis of pancreatic cancer cells. **a** The mRNA level of NOX4 in pancreatic cancer tissues and adjacent normal tissues from GSE15471 and GSE16515. **b** The NOX4 expression of pancreatic cancer samples of different pathologic stages and histologic grades in TCGA database. **c** Kaplan–Meier survival analysis for NOX4 expression in 56 PC patients. **d** The representative IHC section of PC tissues for NOX4 and IHC scores of 56 PC patients for NOX4 corresponding to different tumor grades. HE staining from the same area was used to prove that NOX4 was expressed in tumor cells. **e**, **f** The migration and invasion of HPAC and Panc1 cells after NOX4 overexpress and knockdown by transwell assays. **g** The number of metastatic lesions and percent of metastatic area in the lung section 4 weeks after HPAC cells with NOX4 knockdown and overexpression injection. Four mice for each group. ns, no significance, *p < 0.05, **p < 0.01, ***p < 0.001

To investigate NOX4 expression in PDAC, we analyzed NOX4 expression in GEO databases (GSE15471 and GSE16515). NOX4 expression is upregulated in bulk PDAC tissues compared to normal controls (Fig. 3a). Similar results were found in 7 PDAC tissues compared with 6 benign pancreatic tissues collected in our center (Additional file 3: Figure S2E). We also observed that PDAC cell lines had a higher level of NOX4, as compared to human ductal pancreatic epithelial cells (HPDE cell line, Additional file 3: Figure S2F). By analyzing TCGA data, the expression of NOX4 was significantly correlated with tumor stage and grade (Fig. 3b). We further validated the expression of NOX4 in our clinical cohort and found that PC patients with a higher NOX4 expression had worse overall survival and higher histologic grade than those with lower NOX4 expression (Fig. 3c, d).

This finding prompted us to investigate the functional significance of NOX4. We first performed migration and invasion assays after the knockdown and overexpression of NOX4 in HPAC and Panc1 cells. We found that NOX4 overexpression enhanced the migration and invasion of HPAC and Panc1 cells. However, after the knockdown of NOX4, the migration and invasion were inhibited (Fig. 3e, f). Then, a lung-metastasis xenograft mouse model was established to study the potential role of NOX4 in PC metastasis in vivo. The number of metastatic lesions and percentage of metastatic area were significantly reduced after NOX4 knockdown and increased after NOX4 overexpression in HPAC cells (Fig. 3g). Therefore, these results suggested that NOX4 was upregulated in PC cells and advanced pro-metastatic phenotypes in vitro and in vivo.

### Hypoxia-induced NOX4 activation was independent of HIF1A, but dependent on TGFB1

**Fig. 4:**
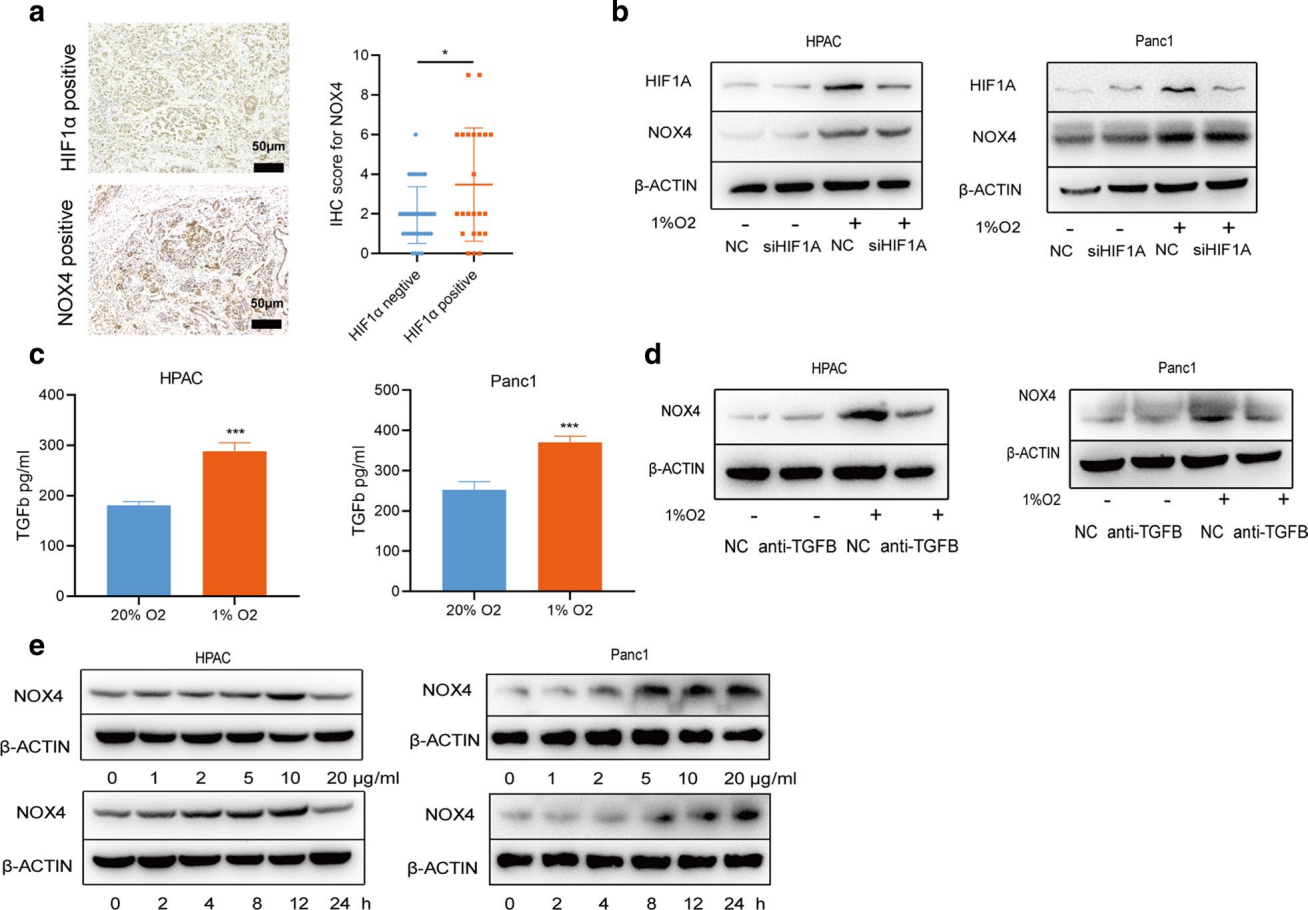
Hypoxia-induced NOX4 activation was independent of HIF1A, but dependent on TGFB1. **a** The representative IHC sections and statistical analysis of NOX4 and HIF1A expression in PC tissues. **b** The protein expression of NOX4 and HIF1A after knockdown of HIF1A and treatment of 1% O2. **c** The secretion of TGFB1 by HPAC cells after hypoxia was determined by ELISA. **d** The protein expression of NOX4 after treatment with TGFB1 neutralizing antibody and/or 1%O2 in HPAC cells. **e** The protein expression of NOX4 after treatment with TGFB1 in time and concentration gradient in HPAC cells. *p < 0.05, **p < 0.01, ***p < 0.001

Since HIF1A is a vital transcription factor induced by hypoxia, we then analyzed whether NOX4 is a downstream gene of HIF1A. Firstly, we compared the IHC scores of NOX4 in HI1FA-positive and HI1FA-negative PC tissues within a cohort of 56 PC patients from our center and found that NOX4 was highly expressed in HI1FA-positive PC tissues (Fig. 4a). However, we found the upregulation of NOX4 preceded to HIF1A induction upon hypoxia exposure (Fig. 2c). And also, the hypoxia-induced NOX4 expression was not affected after the knockdown of HIF1A using siRNA (Fig. 4b). Given that transforming growth factor beta 1 (TGFB1) is a NOX4 inducer [30], we found that TGFB1 secreted by HPAC and Panc1 cells increased significantly after hypoxia (Fig. 4c). TGFB1-neutralizing antibody treatment attenuated hypoxia-induced NOX4 activation in HPAC cells (Fig. 4d). Then, we treated HPAC and Panc1 cells with TGFB1 in time and concentration gradient, and similarly to previous studies, NOX4 is significantly activated by TGFB1 in PC cells (Fig. 4e). Therefore, hypoxia induced NOX4 activation in a HIF1A-independent manner, and TGFB1 may be the upstream signal of NOX4 during hypoxia.

### NOX4 upregulated SNAIL1 expression by increasing histone methylation

**Fig. 5:**
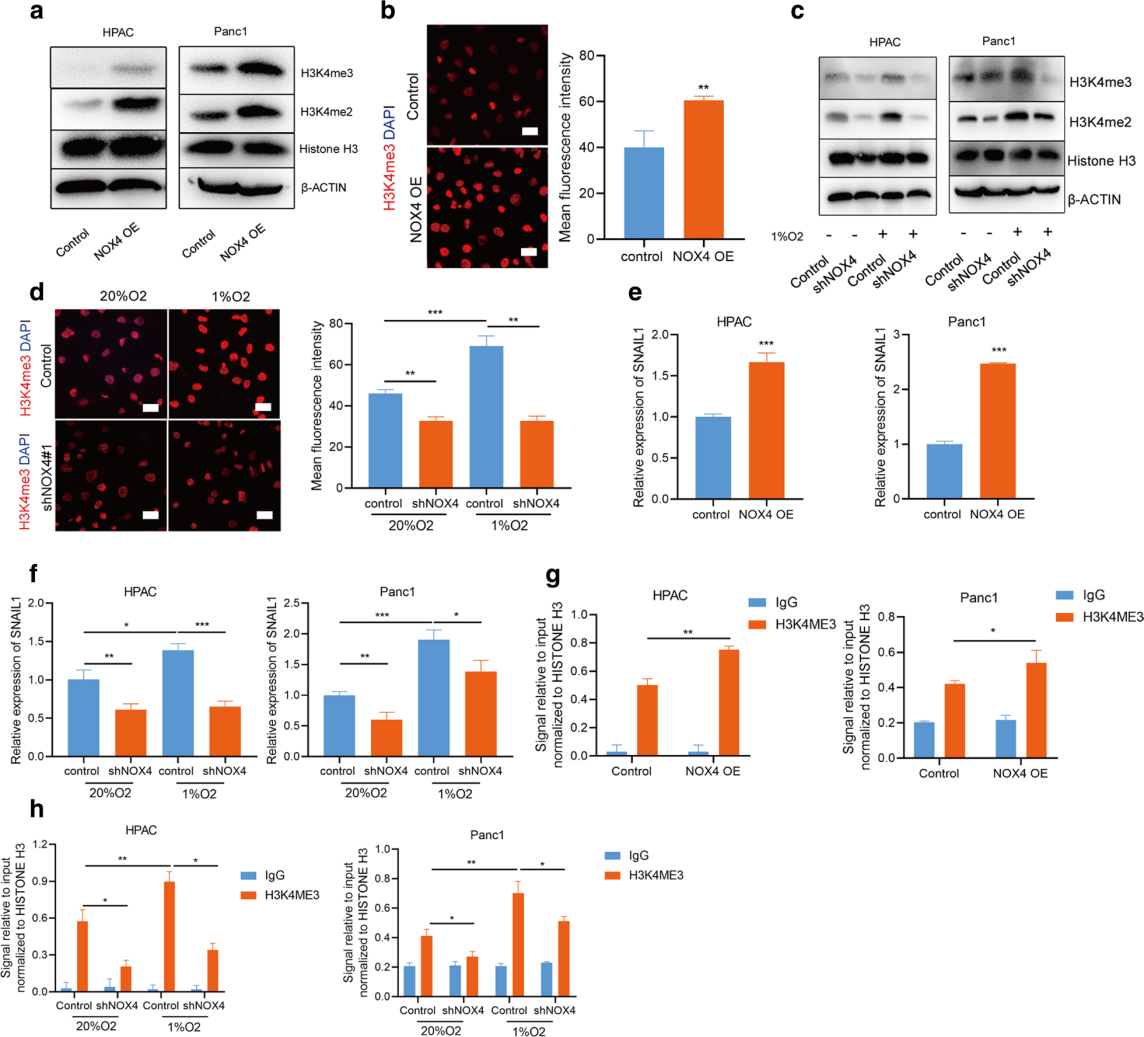
NOX4 upregulated SNAIL1 expression by promoting histone methylation. **a** The protein expression of histone methylation markers after NOX4 overexpression. **b** The expression of H3K4ME3 and the quantification of signal intensities after NOX4 overexpression. Bar = 20 μm. **c** The protein expression of histone methylation markers after NOX4 knockdown with or without of 1%O_2_ treatment. **d** The expression of H3K4ME3 and the quantification of signal intensities after NOX4 knockdown with or without of 1%O_2_ treatment. Bar = 20 μm. **e**, **f** The mRNA level of SNAIL1 in HPAC and Panc1 cells after overexpression and knockdown of NOX4 with or without of 1%O_2_ treatment. **g**, **h** ChIP-qPCR analysis of H3K4me3 for SNAIL1 in HPAC and Panc1 cells after overexpression and knockdown of NOX4 with or without of 1%O_2_ treatment. *p < 0.05, **p < 0.01, ***p < 0.001

The previous study has shown that hypoxia induced a robust increase in histone methylation markers. In particular, chromatin immunoprecipitation followed by deep sequencing (ChIP-sequencing) of H3K4me3, a methylation marker associated with active gene transcription, identified that EMT was upregulated after hypoxia exposure [10]. To investigate whether NOX4 significantly affected histone methylation modifications, we tested the expression of histone methylation markers after overexpressing NOX4. We found that NOX4 induced an increase in histone methylation in HPAC and Panc1 cells (Fig. 5a, b). Then, we focused on the H3K4me3, as we verified the previous findings that the expression of H3K4me3 was indeed significantly induced by hypoxia in HPAC and Panc1 cells (Fig. 5c, d). Compellingly, the knockdown of NOX4 reversed the upregulation of H3K4me3 caused by hypoxia (Fig. 5c, d). Then, we analyzed the ChIP-sequencing results of PC cell line for H3K4ME3 in the GEO database (GSE945856) and found a peak in the promoter region of SNAIL1. Our results confirmed that the mRNA level of SNAIL1 was significantly increased after NOX4 overexpression (Fig. 5e), and knockdown of NOX4 reversed the upregulation of mRNA level of SNAIL1 caused by hypoxia (Fig. 5f). Thus, we performed ChIP-PCR of H3K4me3 at ChIP-sequencing peak for SNAIL1 in HPAC and Panc1 cells after altering the expression of NOX4. The results revealed a marked increase in H3K4me3 at SNAIL1 after NOX4 overexpression or 24 h of hypoxia exposure (Fig. 5g, h). When NOX4 was knocked down with shRNA, it was partially compromised (Fig. 5h). Thus, our results indicated the potential role of NOX4 in promoting methylation of Histone H3 lysine 4 (H3K4) and transcription of SNAIL1, a key transcription factor regulating EMT.

### NOX4 regulated H3K4me3 methylation through promoting the inactivation of KDM5A

**Fig. 6:**
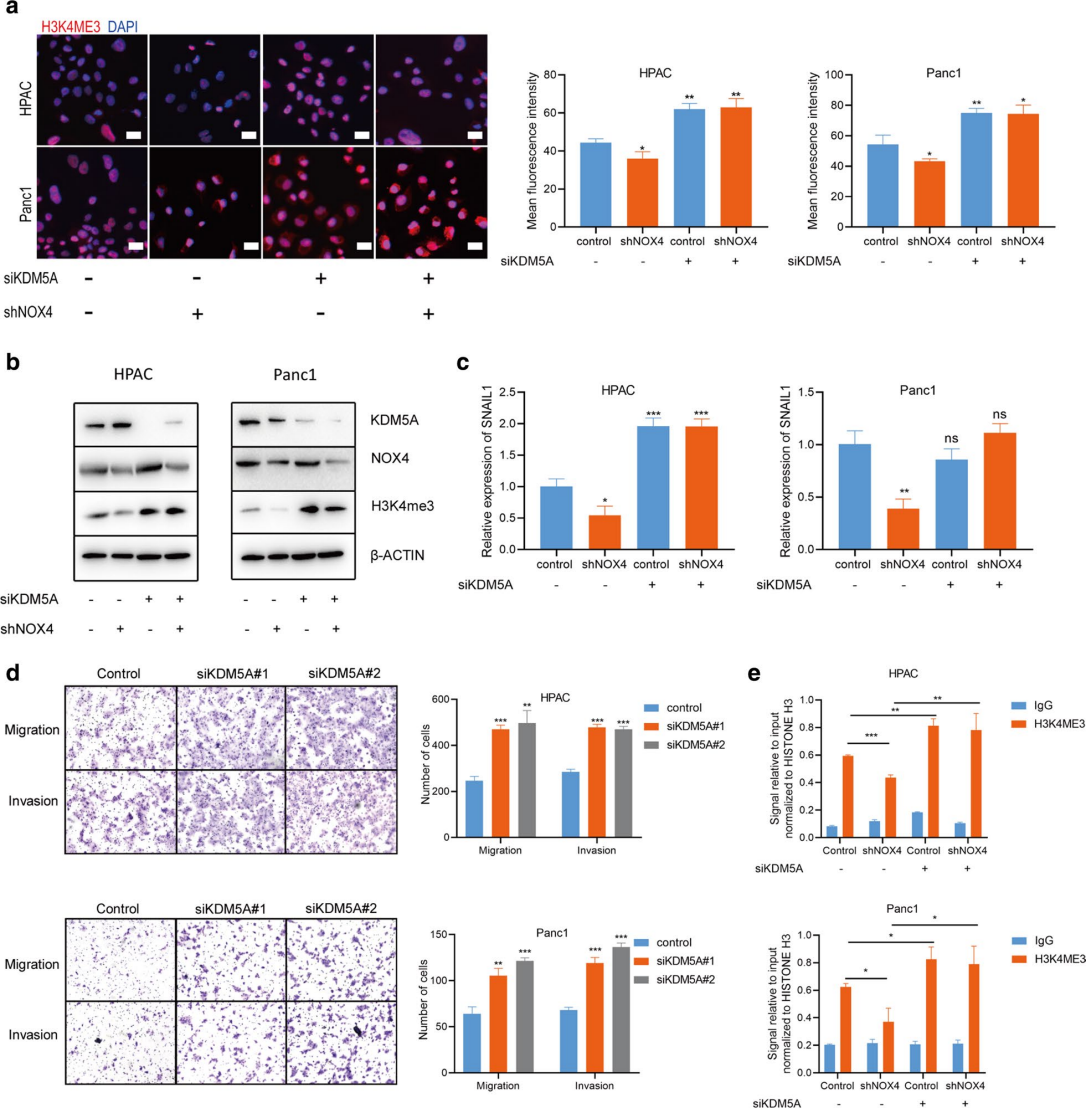
NOX4 regulated H3K4me3 methylation through promoting the inactivation of KDM5A. **a** The expression of H3K4ME3 and the quantification of signal intensities after NOX4 knockdown with or without knockdown of siKDM5A. Bar = 20 μm. **b** The protein expression of histone methylation markers after NOX4 knockdown with or without knockdown of siKDM5A. **c** The mRNA level of SNAIL1 in HPAC and Panc1 cells after NOX4 knockdown with or without knockdown of siKDM5A. **d** The migration and invasion of HPAC and Panc1 cells after knockdown of siKDM5A. **e** ChIP-qPCR analysis of H3K4me3 for SNAIL1 in HPAC and Panc1 cells after NOX4 knockdown with or without knockdown of siKDM5A. ns, no significance, *p < 0.05, **p < 0.01, ***p < 0.001

KDM5A is a kind of jumonji domain-containing demethylase for H3K4ME3, and it has been reported to act as a molecular oxygen sensor in the tumor cells [31]. We further explored whether NOX4 changed the methylation status of histones H3 by affecting the function of KDM5A. Our results showed that knockdown of KDM5A using siRNAs effectively reversed the decrease in H3K4ME3 expression caused by NOX4 knockdown (Fig. 6a, b). Similarly, the mRNA level of SNAIL1 was also significantly reversed after knocking down KDM5A (Fig. 6c). Knockdown of KDM5A also exhibited a significant promoting effect on the invasion and migration of HPAC and Panc1 cells (Fig. 6d). Then, we performed ChIP-PCR of H3K4me3 at ChIP-sequencing peak for SNAIL1 in HPAC and Panc1 cells after altering the expression of NOX4 and KDM5A. We found a marked decrease in H3K4me3 at SNAIL1 after NOX4 knockdown; however, when KDM5A was knocked down, this inhibitory effect was almost reversed (Fig. 6e). These data indicated that NOX4 transcriptionally regulated SNAIL1 to promote the process of EMT by repressing the function of KDM5A and reinstating H3K4ME3 levels.

## Discussion

Hypoxia is an adverse living condition for tumor cells; however, it leads to aggressive phenotypes in a variety of tumors [4, 32, 33]. As we presented, hypoxia-related gene signature was prognostic and linked with upregulated EMT pathway. NOX4-induced oxidative stress and rapid changes in chromatin modification status were necessary processes facilitating this hypoxia-induced EMT process.

We quantified hypoxia score in the TCGA using 30 hypoxia-related genes that are highly expressed in PC and described the correlation between hypoxia score and clinical parameters in these samples. Similar to previous studies in other tumors [34, 35], samples with high hypoxic score exhibited a worse prognosis. Molecular subtypes of PC have been defined by several studies and linked with prognosis and response to treatment [21–23]. Here, we showed that samples with high expression of hypoxia-related genes were more concentrated in the subtype with the worst prognosis (squamous in Bailey clusters, QM in Collisson clusters and basal-like in Moffitt clusters). These results suggested that hypoxia is a driving factor that promoted the transformation of tumors to a more aggressive phenotype.

Hypoxia triggers varied molecular responses in tumor cells. To better understand the potential regulatory mechanism of PC cells under hypoxia, we used hypoxia-related gene signature to predict possible downstream pathways. Here, we found a strong positive correlation between hypoxia-related gene expression and EMT process in PC samples. EMT was induced by hypoxia in a variety of tumors including non-small cell lung cancer (NSCLC) and ovarian carcinoma [36, 37], and the mechanism involves the regulation of SNAIL, twist family bHLH transcription factor (TWIST) and snail family transcriptional repressor 2 (SLUG) [38, 39]. Stabilization of HIF-1a is a crucial transcription factor caused by intratumoral hypoxia, which can induce EMT binding directly to the promoter of TWIST and SNAIL [39, 40]. However, in our results, we found a rapid induction of NOX4 after hypoxia in PC cells, which significantly promotes the EMT process that is independent of activation of HIF1A.

NOX4, as a member of the NADPH oxidase family, is widely expressed in various tumor cells [41] and has been reported to be activated to promote tumor metastasis in some tumors such as human colorectal cancer and non-small cell lung cancer [42, 43]. In PC, Dasgupta et al. found that NOX4 promotes pancreatic cancer-induced cachexia in mice [44]. Our experiments demonstrated NOX4 overexpress or inhibition in pancreatic cancer cells caused changes of invasion and metastatic ability. And these were consistent with the analysis of clinical data from TCGA and our cohort.

Changes in chromatin modification after hypoxia, especially histone methylation, are important newly discovered mechanisms in recent years [10]. This rapid oxygen-sensing mechanism causes a series of critical changes of important pathways to make tumor cells respond to hypoxia. Whether NOX4 could regulate the changes of histone methylation status and epigenetic modification has not yet been reported. We firstly demonstrated that NOX4 could induce stable histone methylation after hypoxia, which lead to the regulation of important pathways including EMT. And this process of oxygen sensing seemed to be earlier than the activation of HIF1A. We also delved into the possible mechanism of NOX4 changing histone methylation status, and our results indicated that KDM5A may be an important mediator for NOX4 to induce increased expression of H3K4ME3.

The limitation of this study is that we did not explore in detail how hypoxia activates NOX4 in a way that does not depend on HIF1A. Although studies have shown that TGFB1 plays an important role in the activation of NOX4 [30] and our research partially illustrates this, the mechanism of how NOX4 remains upregulated under long-term hypoxia conditions has not yet been explained. In addition, how the function of KDM5A is changed by the redox state in the cell also needs to be further explored. One possible explanation is that ROS will oxidize Fe (II) to Fe (III), thereby attenuating the activity of JmjC domain-containing histone demethylases [45].

In summary, we demonstrate for the first time that upregulation of NOX4 after hypoxia can induce histone methylation through altering intracellular ROS levels and promoting the inactivation of KDM5A. Upregulation of histone modifications, especially activation of H3K4ME3, is associated with active gene transcription of SNAIL1, which cause rapid and robust EMT process.

## Supplementary information

**Additional file 1.** Supplementary Information.

**Additional file 2.** **Supplementary Figure 1.** Hypoxia-related gene expression was positively correlated with EMT-related gene expression in pancreatic cancer specimens.

**Additional file 3.** **Supplementary Figure 2.** NOX4 was overexpressed in pancreatic cancer cells and activated EMT pathway.

**Additional file 4.** **Supplementary Table 1.** Hypoxia score of the pancreatic cancer samples in TCGA.

**Additional file 5.** **Supplementary Table 2.** EMT score of the pancreatic cancer samples in TCGA.

**Additional file 6.** **Supplementary Table 3.** Genes positively related to hypoxia score using pearson correlation analyse in TCGA PAAD samples.

## Data Availability

Data sharing is not applicable to this article as no datasets were generated during the current study.

## Abbreviations

PC: Pancreatic cancer
NOX: NADPH oxidase
KDM5A: Lysine demethylase 5A
EMT: Epithelial-to-mesenchymal transition
SNAIL1: Snail family transcriptional repressor 1
HIF1A: Hypoxia-inducible factor 1 subunit alpha
ROS: Reactive oxygen species
GO: Gene Ontology
GSEA: Gene-set enrichment analysis
FFPE: Formalin-fixed paraffin-embedded
CHIP: Chromatin immunoprecipitation
OS: Overall survival (OS)
PFS: Progression-free survival
QM: Quasimesenchymal
VIM: Vimentin
CDH2: Cadherin 2
CDH1: Cadherin 1
TGFB1: Transforming growth factor beta1
NSCLC: Non-small cell lung cancer
TWIST: Twist family bHLH transcription factor
SLUG: Snail family transcriptional repressor 2
