# Supplementary material for: Hypoxia promotes the metastasis of pancreatic cancer through regulating NOX4/KDM5A-mediated histone methylation modification changes in a HIF1A-independent manner
Source: Clin Epigenetics. 2021 Jan 26;13:18. doi: 10.1186/s13148-021-01016-6 (PMC7836598; doi:10.1186/s13148-021-01016-6)
Supplement: Supplementary file 1 — Additional file 1. Supplementary Information. [file 13148_2021_1016_MOESM1_ESM.docx]

**Supplementary Information**

**Supplementary Figure S1. Hypoxia-related gene expression was positively correlated with EMT-related gene expression in pancreatic cancer specimens.** (A) Expression profiles of 30 hypoxia-related genes across pancreatic cancer samples in TCGA. (B) Hypoxia related GO biological processes and (C) Hallmark gene sets were enriched and visualized. p<0.05. (D) Expression profiles of 200 EMT markers were shown across pancreatic cancer samples in TCGA.

**Supplementary Figure S2.** **NOX4 was overexpressed in pancreatic cancer cells and activated EMT pathway.** (A) GSEA analysis of genes positively related to NOX4 expression showed enriched pathways associated with EMT and hypoxia. (B) The expression of NOX4 in PC tissues was determined by immunohistochemistry. Bar=25μm.(C) Intracellular ROS level and quantification of signal after 1%O2 treatment in HPAC cells was measured by fluorescence assay. (D) Intracellular ROS level and quantification of signal after NOX4 overexpression in HPAC cells was measured by fluorescence assay. (E) The protein expression of NOX4 in human PC tissues and benign pancreatic lesions (F) The protein expression of NOX4 in human ductal pancreatic epithelial cells and human PC cells. *P < 0.05, **P < 0.01, *** P < 0.001.

**Supplemental Methods and Materials**

**Immunoblot analysis**

Cell or tissue homogenate were washed twice using ice-cold PBS and lysed in RIPA buffer (Beyotime, Shanghai, China) enriched with Cocktail (Roche Diagnostics) and PMSF (Beyotime) for 30 minutes. Then the lysate was collected using 1.5 ml fresh tubes and centrifuged at 14,000 g for 15 minutes. The concentration of supernatant was determined using BCA Protein Assay kit (Beyotime) following the manufacturer’s instructions. 30-40 ug protein were loaded in 10% SDS-PAGE for electrophoresis and then transferred to PVDF membranes. After that, the membranes were blocked with 5% skimmed milk in TBS solution and incubated with primary antibodies (see below) overnight at 4°C. Membranes were washed 3 times (each for 10 min) with 0.05% Tween-20-TBS and were incubated with a horseradish peroxidase (HRP)-conjugated secondary antibody (1:5000) for 2h at room temperature (supplementary material). The protein signals were visualized using an ECL kit (Millipore, Billerica, MA, USA) with a CCD camera (Tanon, Shanghai, China).

**Immunohistochemistry**

Sections were cut from FFEP tissues (4μm thick). Then sections were deparaffinized with xylene and rehydrated in using graded ethanol. The further staining steps were proceeded in accordance with the manufacturer's protocol of Immunohistochemistry Kit (KIT-9720, MXB Biotechnologies, China). Antigen retrieval was carried out in citrate buffer (10Nm, pH 6.0) after high pressure heating for 15 min. Sections were incubated overnight with the NOX4 antibody (proteintech, 14347-1-AP, Rosemont, USA) or HIF1A antibody (Abcam, ab16066, MA, USA). The staining was observed using DAB kit (DAB-0031, MXB Biotechnologies, China). All sections were evaluated by two independent pathologists without knowing the clinical information of the patient. The proportion of stained areas was evaluated as follows: 0, <5%; 1, ≥5% and <25%; 2, ≥25% and <50%; 3, ≥ 50% and <75%; 4, ≥75%. The intensity of staining was scored as follows: 0, negative; 1, weak; 2, medium; 3, strong. The final scores were obtained by multiplying the area and intensity scores, producing a range of 0-12.

**CHIP-PCR**

Protein was cross-linked to chromatin by 1% formaldehyde for 10min at 37°C and 0.125 M glycine was used to stop crosslinking. Then cells were washed twice in DPBS, harvested in ChIP lysis buffer and sonicated. supernatants were collected (input). Then samples were diluted in dilution buffer, incubated with antibodies or IgG overnight at 4°C and captured using protein A/G magnetic beads (MCE, Shanghai, China). Immunoprecipitates were washed using Wash Buffer 1, Wash Buffer 2, Wash Buffer 3 and TE buffer (twice) and then eluted with 120μL of Elution Buffer. Crosslinks were reversed using 0.2M NaCl overnight at 65℃ and proteins were digested by 40mM Tris-HCl (pH6.5), 10mM EDTA and 20μg Proteinase K for 1h at 45°C. DNA was purified and used for RT-PCR.

The buffer formulations were as follows: Wash Buffer 1: 20mM Tris-HCl (pH 8.1), 0.1% SDS, 1% Triton X-100, 2mM EDTA and150mM NaCl; Wash Buffer 2: 20mM Tris-HCl (pH 8.1), 0.1% SDS, 1% Triton X-100, 2mM EDTA and 500mM NaCl; Wash Buffer 3: 10mM Tris-HCl (pH 8.1), 0.25M LiCl, 1% (v/v) NP-40, 1% Na-deoxycholate and 1mM EDTA; TE Buffer: 10mM Tris-HCl (pH 8.0) and 1mM EDTA; Elution Buffer: 1% SDS and 0.1M Na-bicarbonate. The primer sequences used were shown in supplementary material.

**TGF-beta ELISA**

The supernatant of HPAC cells with or without 1% O2 treatment for 24 hours was used to detect the secretion of TGF-beta according to the manufacturer's instructions (MULTISCIENCES BIOTECH, CO., LTD, Hangzhou, China).

**Cell treatment**

HPAC cells were treated with TGF-beta 1 (R&D systems, MI, USA) with the concentration of 0, 1, 2, 5, 10, 20μg/ml for 24 hours or with the concentration of 10μg/ml for 0, 2, 4, 8, 12, 24 hours, then cells are used for Immunoblot analysis. HPAC cells were treated with TGF-beta 1 antibody (R&D systems) with the concentration of 1μg/ml for 24 hours, then cells are used for Immunoblot analysis.

**HIF1A and KDM5A knockdown**

HIF1A and KDM5A siRNAs or scrambled control siRNA were designed and synthesized from HANBIO (Shanghai, China). Cells were plated into six-well plates (3×105 cells per well). Before transfection, the culture medium was replaced with DMEM with 10%FBS. SiRNA and negative control siRNA were incubated with iMAX (Thermo Fisher Scientific) in OPTIMEM medium for 15 minutes, and then transferred to DMEM medium for 72 hours.

**Sequences of primers for quantitative RT-PCR and CHIP-PCR**

| **Gene** | **Sequence 5′-3′** | |
| --- | --- | --- |
|  | **forward** | **reverse** |
| **RT-PCR** |  |  |
| NOX4 | GTCTTTGACCCTCGGTCCTC | GAGTGTTCGGCACATGGGTA |
| VIM | TCCGCACATTCGAGCAAAGA | ATTCAAGTCTCAGCGGGCTC |
| CDH1 | GGGGTCTGTCATGGAAGGTG | CAAAATCCAAGCCCGTGGTG |
| CDH2 | ATGGGAAATGGAAACTTGATGGC | TGGAAAGCTTCTCACGGCAT |
| SNAIL1 | CGAGTGGTTCTTCTGCGCTA | GGGCTGCTGGAAGGTAAACT |
| ACTB | CTCGCCTTTGCCGATCC | TCTCCATGTCGTCCCAGTTG |
| **CHIP-PCR** |  |  |
| SNAIL1 | AGAACCAGGGGAGGACGATT | GGCCTCGTAGGAGTTTGGAC |
| SNAIL1 control | CTCGGGCCTTTTCCCTTGAT | TTGACGAGGGAAACGCACAT |

**Antibodies for western blot, immunohistochemistry and immunofluo-rescence**

| **Antibody** | **Vendor** | **Catalog No.** |
| --- | --- | --- |
| **Primary antibody** |  |  |
| anti-ACTIN | Sigma | A3854 |
| anti-NOX4 | Proteintech | 14347-1-AP |
| anti-CDH1 | Abcam | ab1416 |
| anti-CDH2 | Santa Cruz | sc7939 |
| anti-HIF1A | Abcam | ab16066 |
| anti-SNAIL1 | Cell Signaling Technology | 3879s |
| Anti-VIM | Proteintech | 10366-1-AP |
| anti-H3K4ME3 | Cell Signaling Technology | 9751s |
| anti-HISTONE H3 | Cell Signaling Technology | 4499s |
| anti-H3K4ME2 | PTM BIO | PTM-641 |
| anti-H3K9ME3 | PTM BIO | PTM-616 |
| anti-H3K27ME2 | PTM BIO | PTM-621 |
| anti- H3K27ME3 | PTM BIO | PTM-622 |
| anti- H3K36ME2 | PTM BIO | PTM-624 |
| anti- H3K36ME3 | PTM BIO | PTM-625 |
| Secondary Antibody |  |  |
| Anti-rabbit IgG, HRP-linked Antibody | Cell Signaling Technology | 7074s |
| Anti-mouse IgG, HRP-linked Antibody | Cell Signaling Technology | 7076s |
| Donkey Anti-Rabbit IgG H&L (DyLight® 594) | Abcam | ab96921 |
| Fluorescein (FITC) AffiniPure Goat Anti-Mouse IgG | Jackson ImmunoResearch | 115-095-071 |
