# Supplementary figures and images for: Hypoxia promotes the metastasis of pancreatic cancer through regulating NOX4/KDM5A-mediated histone methylation modification changes in a HIF1A-independent manner
Source: Clin Epigenetics. 2021 Jan 26;13:18. doi: 10.1186/s13148-021-01016-6 (PMC7836598; doi:10.1186/s13148-021-01016-6)

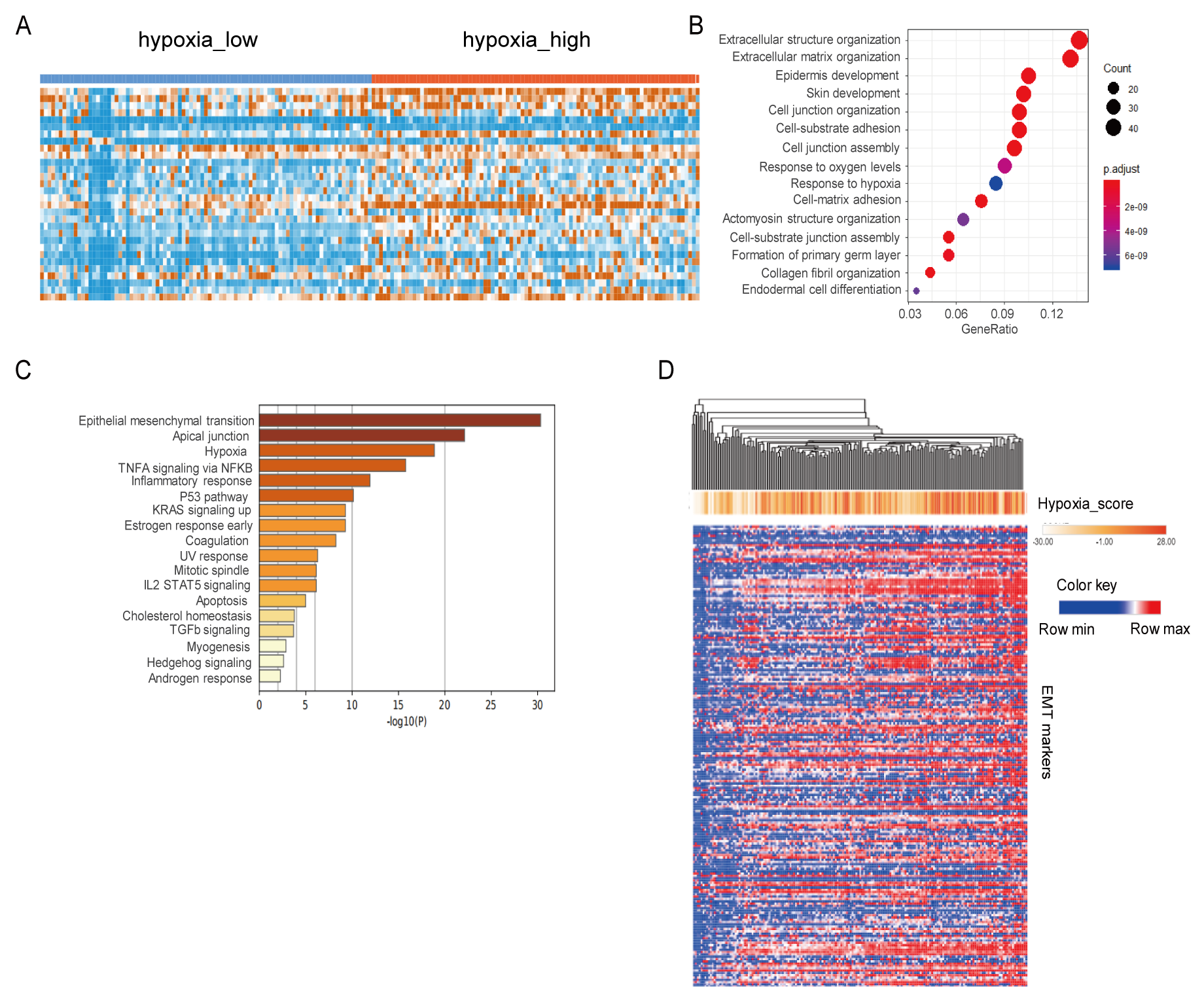

Supplement: Supplementary file 2 — Additional file 2. Supplementary Figure 1. Hypoxia-related gene expression was positively correlated with EMT-related gene expression in pancreatic cancer specimens. [file 13148_2021_1016_MOESM2_ESM.tif]

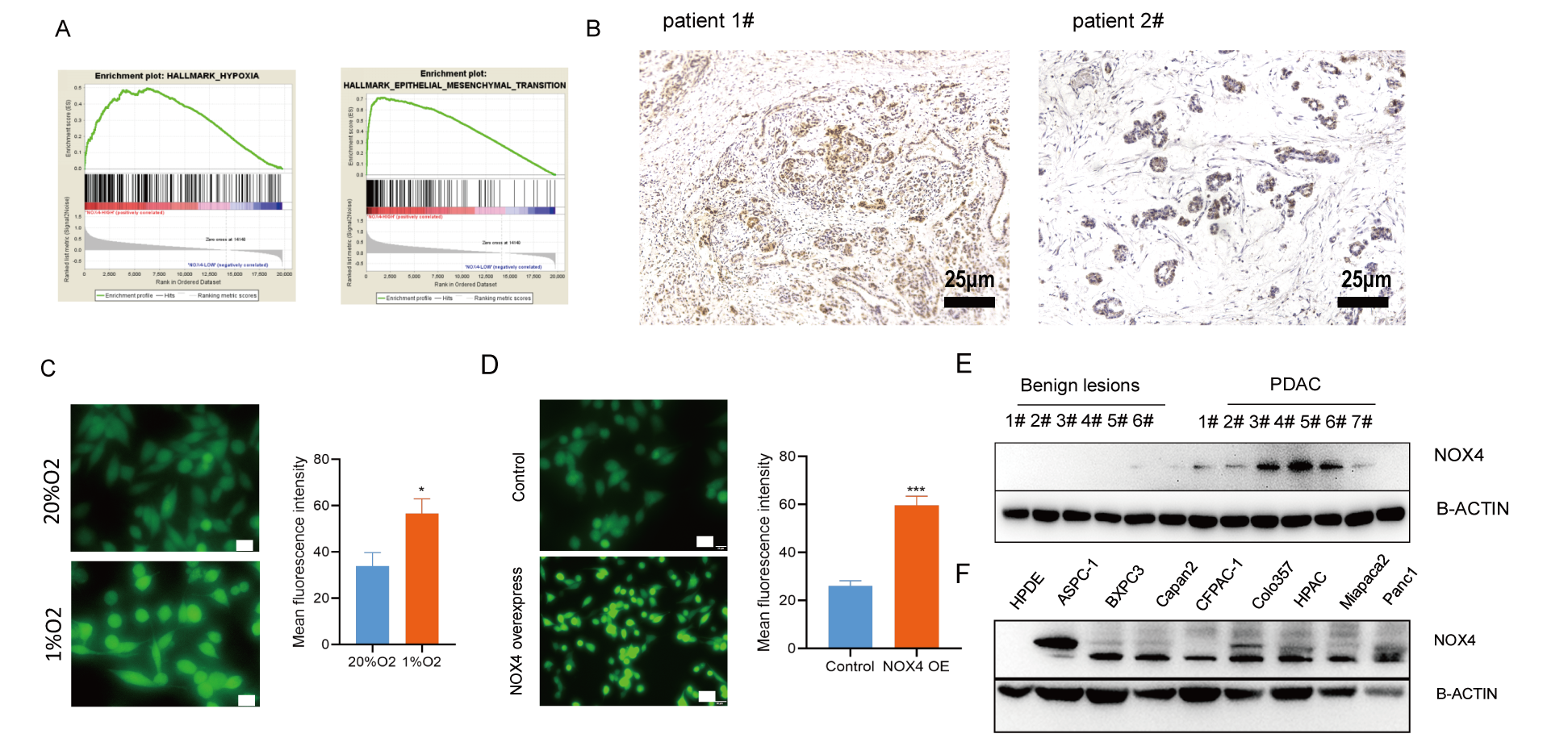

Supplement: Supplementary file 3 — Additional file 3. Supplementary Figure 2. NOX4 was overexpressed in pancreatic cancer cells and activated EMT pathway. [file 13148_2021_1016_MOESM3_ESM.tif]
